# Visual Sensor Networks and Related Applications

**DOI:** 10.3390/s19224960

**Published:** 2019-11-14

**Authors:** Daniel G. Costa, Francisco Vasques, Mario Collotta

**Affiliations:** 1 Department of Technology, State University of Feira de Santana, Feira de Santana 44036-900, Brazil; 2 Faculty of Engineering, University of Porto, 4200-465 Porto, Portugal; vasques@fe.up.pt; 3 Faculty of Engineering and Architecture, Kore University of Enna, 94100 Enna, Italy; mario.collotta@unikore.it

## 1. Overview

The use of sensing devices to perform monitoring tasks has continuously evolved in the past decades. The initial application scope has been enlarged and connected sensors have become common in many monitoring scenarios. In this process, new communication standards and hardware platforms have been developed to support sensor-based applications, bringing the distributed sensing paradigm to its current spotlight.

The maturation of the sensor networks has happened following different but sometimes concurrent paths, associating different areas of Computer Science and Engineering. One of such paths has exploited camera-based sensors to retrieve visual data, opening new and exciting perspectives for this area. The resulting Visual Sensor Networks have been an important research topic due to the large complexities associated to the capturing, processing, transmission, and storage of images and videos, as well as the wide applicability of such visual data for different monitoring and control functions.

There are many challenges when employing visual sensor networks. The large amount of data produced by visual sensors will inevitably raise energy and bandwidth concerns, which may severely impact the entire network operation. Moreover, many applications may have real-time delivery demands, which may be hard to accomplish in ad hoc sensor networks. Finally, security issues as confidentiality and authenticity put additional processing burden on some sensors, which will be already striving to handle visual data. All these complex scenarios have fostered research works to optimize and enhance visual sensor networks.

Besides addressing the challenging issues related to the visual sensors operation cycle, research works have been concerned with the adoption of cameras as sensing units, improving traditional sensor-based applications and even fostering the development of new ones. Actually, the evolving Internet of Things landscape has taken visual sensor nodes as an important source of information, directly benefiting applications in traffic control, public security, industry 4.0, vehicular networks, and smart agriculture, among many others. For the coming years, new possibilities for visual sensing are still expected.

This Special Issue presents innovative research papers addressing classical and new challenges of visual sensor networks and related applications. A total of eight papers were submitted for it, but only five papers were accepted for publication. The published papers bring relevant contributions to the area, being an important reference for new works. In this sense, we would like to thank all authors who have submitted their manuscripts to this Special Issue and also the reviewers for their hard work during the review process.

## 2. Summary of the Special Issue

The published works bring important contributions to the area of visual sensor networks, but addressing different aspects of those networks and their related applications. In fact, the five works of this special issue have different impacts on this research area: while some works bring more direct contributions for optimizations, enhancement, and practical use of visual sensor networks, as in [1,2,3]; the other works bring contributions that can indirectly benefit the construction of visual sensor networks and related applications, as in [4,5]. All these contributions are briefly described as follows.

In [1], authors propose a new low-cost and low-power visual sensor platform based on the Arduino open-source board and additional components. This practical platform is motivated by the need to efficiently perform block compressive sensing on visual data in order to reduce the amount of data to be transmitted by the nodes, since energy and transmission efficiency are highly desired for visual sensor networks. For the compressive sensing paradigm, most complexity is left for the receiver side, reducing overload on the sensor nodes. As the visual sensor platform was created from scratch, employing off-the-shelf components, authors could implement the proposed compressive sensing method in a more efficient way.

In [2], the silicon retinas (Dynamic Vision Sensor—DVS) are considered as a special kind of visual sensor. In this defined context, since such sensors will transmit observed “scenes” instead of traditional video streams, the bandwidth demands for such configuration should be properly known and optimized. This work exploits the silicon retinas as a suitable candidate for next-generation visual sensor networks, investigating the relation of different parameters of scene-capturing with the transmission of visual data, achieving some promising results.

The work in [3] surveys communication protocols with QoS guarantees for wireless sensor networks deployed for multimedia sensing. Recent works covering new developments in this area are discussed, focusing on four pillars: energy efficiency, real-time data delivery, transmission of large volume of sensory data, and directional sensor coverage. Comparisons among the surveyed works are also performed, potentially supporting new works in this research area.

In [4], the problem of device-to-device (D2D) communications is investigated, focusing on cellphones. The authors want to optimize communications between close cellphones, addressing the increasing demand for (large) data transmissions, for example, avoiding the repeated downloads of the same data. For that, a deep neural network is proposed to handle this problem, particularly reducing interference between links and assuring better QoS (Quality of Service) guarantees for the communications.

Finally, in [5], authors propose a new method to perform accurate and real-time prediction of traffic flow. For that, temporal and spatial parameters are considered, which are processed by the proposed AutoEncoder Long Short-Term Memory (AE-LSTM) prediction method. Actually, authors demonstrate a more efficient way to predict traffic, which is highly desired in intelligent transportation systems in the scope of smart cities. In addition, many of the current systems are employing visual sensors to retrieve real-time data about the vehicles’ flows, allowing easier integration of the proposed method to practical applications.
